# Effective management of SMARCA4-deficient undifferentiated tumor by immunotherapy combined with chemotherapy and radiotherapy: Case report

**DOI:** 10.1097/MD.0000000000045747

**Published:** 2025-10-31

**Authors:** Xin Liang, Tao Zheng, Qingyu Ge, Rong Huang

**Affiliations:** a Department of Oncology, Fengjie People’s Hospital, Chongqing, China; b Department of Thoracic Surgery, Fengjie People’s Hospital, Chongqing, China.

**Keywords:** chemotherapy, immunotherapy, radiotherapy, SMARCA4-UT, tislelizumab

## Abstract

**Rationale:**

SMARCA4-deficient undifferentiated tumor (SMARCA4-UT) has emerged as a rare subtype of thoracic tumor, whose prognosis is unfavorable and for which standard therapeutic regimen is presently unavailable.

**Patient concerns:**

This report details the case of a male thoracic SMARCA4-UT patient 60 years of age, who lacked the opportunity for surgery. Genomic analysis revealed a significant tumor mutational burden (15.95 muts/Mb).

**Diagnoses:**

The findings of computed tomography, histopathology, immunohistochemical staining, and genomic profiling led to the confirmation of a thoracic SMARCA4-UT diagnosis.

**Interventions:**

The first-line treatment consisted of combination chemotherapy (carboplatin and etoposide), thoracic radiotherapy, and tislelizumab immunotherapy. The second-line treatment included radiotherapy for the right adrenal gland metastasis, combination chemotherapy (paclitaxel and cisplatin), and tislelizumab immunotherapy.

**Outcomes:**

As of July 2025, the patient’s overall survival has exceeded 33 months.

**Lessons:**

Our case of SMARCA4-UT exhibiting a high tumor mutational burden demonstrated a favorable response to the immunotherapy (tislelizumab) combined with chemotherapy and radiotherapy. This approach may represent a novel therapeutic strategy for SMARCA4-UT population.

## 1. Introduction

In 2015, a new category of aggressive thoracic malignancies was reported by Le Loarer et al,^[1]^ designating it as SMARCA4-deficient thoracic sarcomas. The World Health Organization Classification of Thoracic Tumors 5th Edition, which was released by the International Agency for Research on Cancer in May 2021, categorizes this condition as thoracic SMARCA4-deficient undifferentiated tumor (SMARCA4-UT).^[2]^ It is a rare malignancy that primarily affects the chest region in adults, presenting with either a rhabdoid or undifferentiated phenotype and exhibiting BRG1 deficiency.^[2]^ In general, SMARCA4-UT is linked to mutations associated with smoking, including TP53, STK11 and KRAS.^[3,4]^ Currently, no optimal standard therapeutic plan is available for this condition due to a lack of evidence. SMARCA4-UT patients respond poorly to conventional radiotherapy and chemotherapy, or surgery alone, displaying median durations of survival ranging from 4 to 7 months.^[2]^ Furthermore, the absence of common driver gene mutations renders SMARCA4-UT insensitive to targeted therapies. Reports suggest that immunotherapy may represent a promising treatment technique for SMARCA4-UT.^[5,6]^ However, a study shows that this condition primarily has an immune-desert tumor microenvironment, which restricts with limited the effectiveness of immunotherapy.^[7]^ In this report, we describe a male patient with thoracic SMARCA4-UT responding favorably to immunotherapy (tislelizumab) combined with chemotherapy and radiotherapy.

## 2. Case presentation

**Figure 1.**
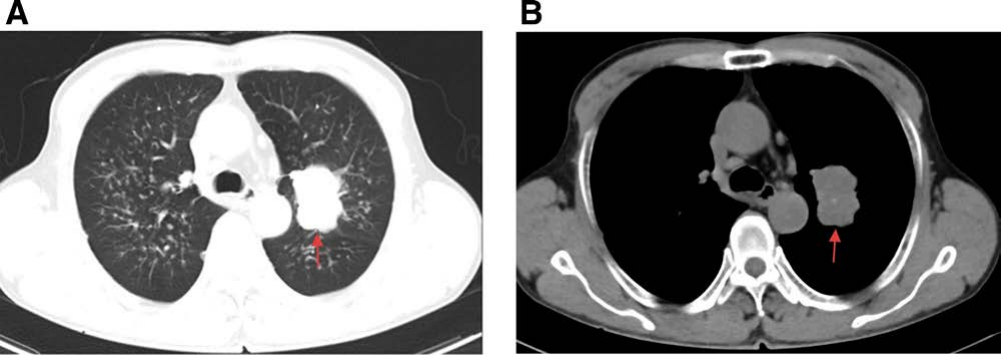
Axial CT images of the pulmonary (A) and mediastinal (B) windows reveal a soft tissue mass located in the left upper lobe of the patient’s lung. CT = computed tomography.

**Figure 2.**
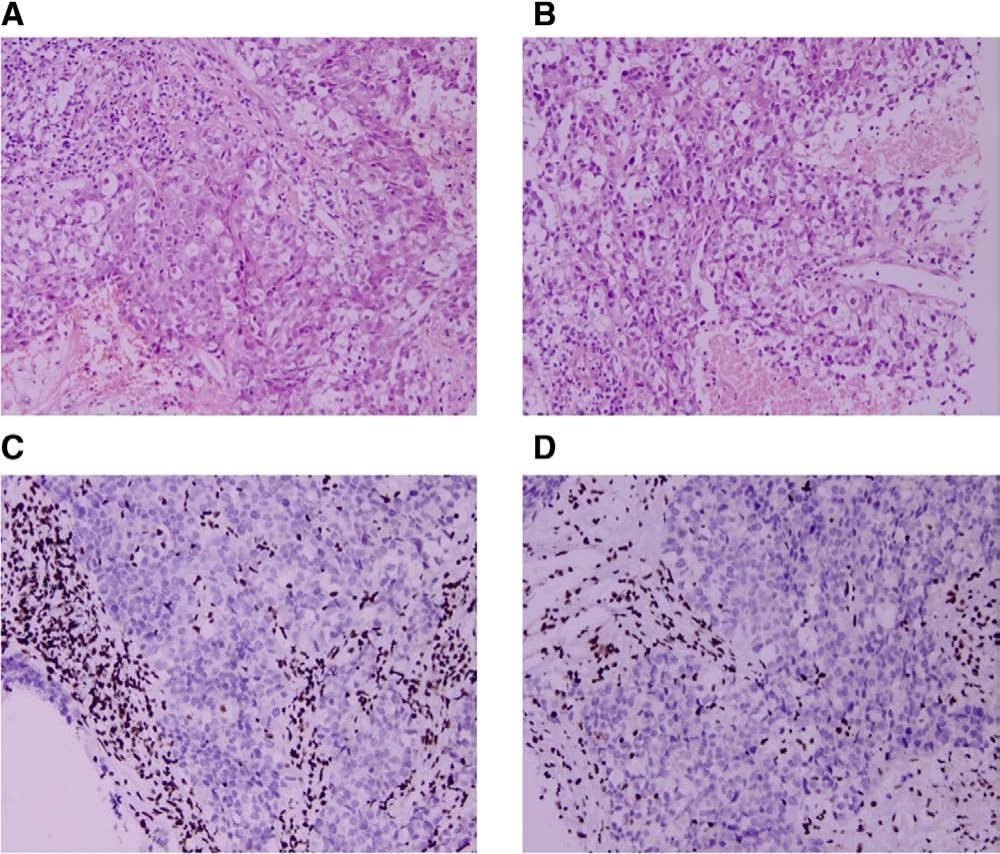
Pathological traits of SMARCA4-UT. (A and B, microscopic magnification × 200) Stained with hematoxylin and eosin. (C and D, microscopic magnification × 200) SMARCA4 (BRG1) negative expression. SMARCA4-UT = SMARCA4-deficient undifferentiated tumor.

**Figure 3.**
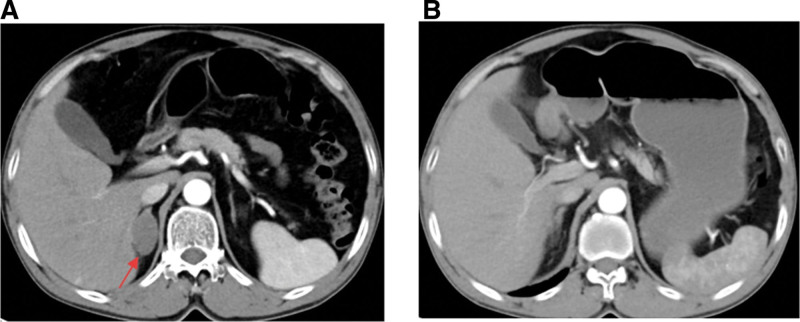
Abdominal CT images demonstrating the disappearance of the right adrenal gland metastasis (A and B). CT = computed tomography.

**Figure 4.**
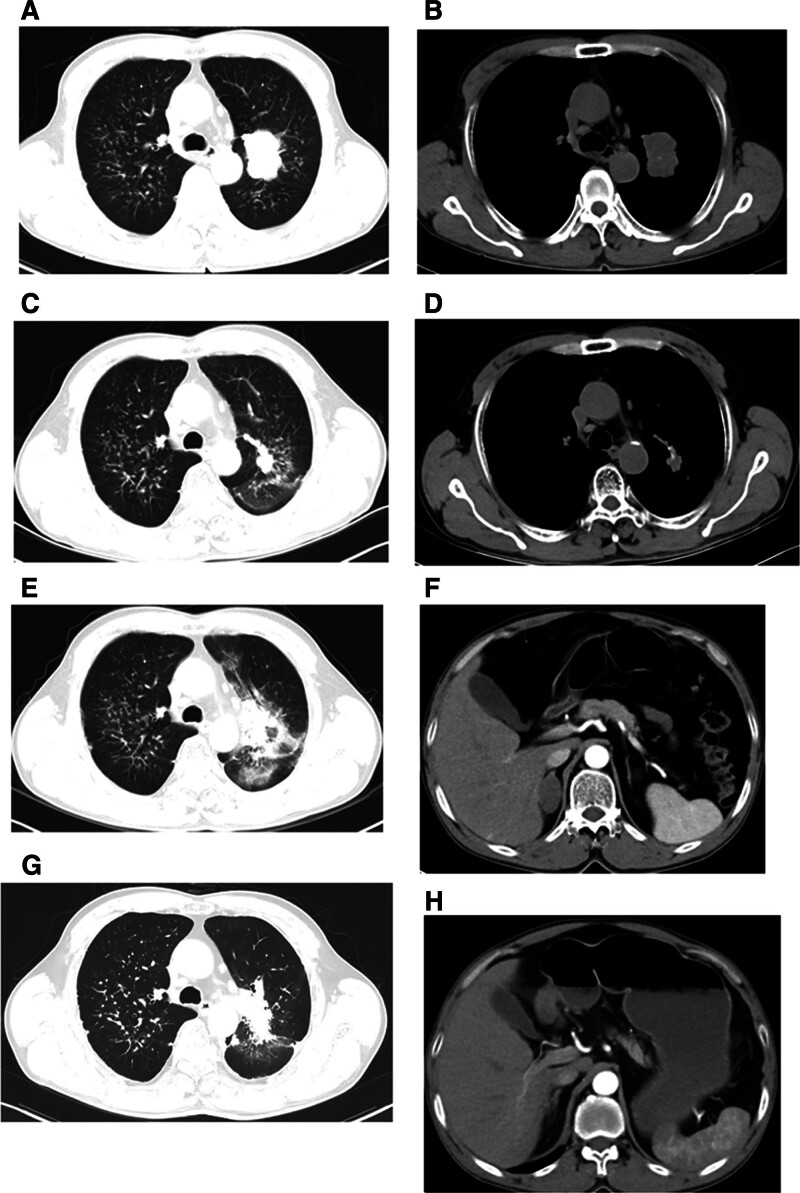
Timeline of the clinical course of the patient. (A and B) Baseline exam. (C and D) Partial response to first-line treatment. (E and F) Disease progression. (G and H) Partial response to second-line treatment.

A Chinese male 60 years of age visited Chongqing Southwest Hospital in October 2020 due to symptoms of cough and expectoration. His past medical and family histories were largely unremarkable. Personal history revealed that he was an active smoker with a recorded history of 40 pack-years. A solid mass measuring 44 mm × 33 mm was identified in the left upper lobe of his lung (Fig. 1) by chest computed tomography (CT) examination, alongside potential multiple metastases in both lungs, although no mediastinal or hilar lymphadenectasis was noted. Contrast-enhanced cranial magnetic resonance imaging, neck, abdomen and pelvis CT scans, as well as bone scan, all failed to reveal distant metastasis. Subsequently, CT-guided percutaneous intrapulmonary needle biopsy was performed. Figure 2A and B illustrate hematoxylin-eosin staining of the mass located in the left upper lobe of the lung. Immunohistochemical staining results: CK(+), Vim(−), S-100(−), AR(−), CK7(−), TTF-1(+), BRG1(−) (Fig. 2C and D), P63(+), Syn(+), Ki-67(+, 80%), BRM(−), INI-1(−), SALL4(+), SOX-2(+), CD34(+), P53(++). To identify potential actionable mutations, 520 cancer-related genes were detected through tumor biopsy samples. Genomic profiling indicated a high tumor mutational burden (TMB) (15.95 muts/Mb), with the tumor demonstrating microsatellite stability. A nonsense mutation in exon 6 of the SMARCA4 gene, along with 6 other mutations (FANCM, TP53, CDK12, ERBB2, RICTOR, and YES1) were considered clinically relevant variants (Table 1). The above findings led to the confirmation of a thoracic SMARCA4-UT diagnosis, and the tumor-node-metastasis staging was classified as clinical T4N0Mx. Accordingly, he was treated with a cycle of combined etoposide and carboplatin as the first-line treatment. Following this, for unspecified reason, the patient chose our hospital for further treatment. He continued to receive 4 cycles of combination chemotherapy comprising carboplatin (AUC = 5, D1) plus etoposide (100 mg/m^2^, D1–3), and subsequent thoracic radiotherapy (60 Gy/30 fractions/6 weeks). During the radiation treatment, etoposide and carboplatin were administered in a single infusion. Radiation pneumonitis developed after radiotherapy. On June 7th, 2023, we initiated the infusion of anti-programmed cell death protein-1 antibody tislelizumab (200 mg, D1, Q3W) to this patient, which was well tolerated without any adverse events. The chest CT scan conducted on August 30, 2023 indicated a partial response to the first-line treatment. Sixteen months later, his CT scan demonstrated disease progression (a metastatic tumor in the right adrenal gland measuring 38 mm × 21 mm), which was corroborated by positron emission tomography/CT at Chongqing Southwest Hospital. Local radiotherapy (45 Gy/15 fractions/3 weeks) was delivered to the right adrenal gland metastasis, and systemic treatment comprising cisplatin (75 mg/m^2^, D1), paclitaxel (135 mg/m^2^, D1), and tislelizumab (200 mg, D1) was given for 2 cycles. Strikingly, an abdominal CT scan posttreatment showed the disappearance of the right adrenal gland metastasis (Fig. 3). Subsequently, tislelizumab was continued as the maintenance treatment and the condition remained stable. The thoracic SMARCA4-UT was initially diagnosed in October 2022, and at the most recent follow-up in July 2025, the patient was still alive. Figure 4 provides a timeline of his clinical course.

**Table 1:** Next-generation sequencing results for gene mutations identified in the tumor biopsy specimen.

Gene	Variation type	Amino acid change	Exon	cDNA change	Mutation abundance
FANCM	Nonsense mutation	p. Glu683*	12	c.2047G>T	50.87%
SMARCA4	Nonsense mutation	p. Glu371*	6	c.1111G>T	53.67%
TP53	Missense mutation	p. Arg273Leu	8	c.818G>T	51.53%
CDK12	Copy number amplification	–	–	17q12	CN:14.0
ERBB2	Copy number amplification	–	–	17q12	CN:14.2
RICTOR	Copy number amplification	–	–	5p13.1	CN:4.3
YES1	Copy number amplification	–	–	18p11.32	CN:4.1

## 3. Discussion

BRG1, one of the switch/sucrose nonfermentable complex members, is encoded by SMARCA4 genes, and possesses tumor-suppressive properties.^[8,9]^ Alterations in SMARCA4 lead to the BRG1 expression loss, thereby facilitating the malignancy growth.^[10]^

Thoracic SMARCA4-UT is an uncommon yet aggressive neoplasm that predominantly afflicts heavy male smokers. Most individuals are diagnosed at a late stage, resulting in an unfavorable prognosis. The median overall survival (OS) of thoracic SMARCA4-UT ranges from 4 to 7 months.^[2,11,12]^ Concerning treatment, there are presently no established guidelines for thoracic SMARCA4-UT. Patients with SMARCA4-UT face a significant risk of relapse post-surgery, even among stage I.^[13]^ For several SMARCA4-UT patients who received a diagnosis at the later stages, neither conventional radiotherapy nor chemotherapy is effective.^[14]^ Mutations in ALK, EGFR or ROS1 are infrequently linked to SMARCA4-UT,^[12,15,16]^ rendering the majority of patients inappropriate for targeted therapies. A recent investigation^[7]^ indicated that a low rate of immune checkpoint inhibitor response in SMARCA4-UT correlates with a lack of tumor-infiltrating lymphocytes within the tumor microenvironment. However, numerous studies conducted over the past few years have underlined the effectiveness of immunotherapy, both as a standalone treatment and in conjunction with chemotherapy, in the SMARCA4-UT population.^[14,17–22]^ Among the strategies of immunotherapy, the reported regimens include: monoimmunotherapy (e.g., nivolumab, pembrolizumab and tislelizumab),^[5,6,18]^ combined immunotherapy (ipilimumab combined with pembrolizumab),^[23]^ immunotherapy combined with chemotherapy (e.g., TEC [tislelizumab plus etoposide and carboplatin], TEP [tislelizumab plus etoposide and cisplatin], ABCP [atezolizumab plus bevacizumab, carboplatin, and paclitaxel)],^[14,21,24]^ and immunotherapy combined with anti-angiogenic agent (pembrolizumab combined with anlotinib, sintilimab combined with anlotinib).^[25,26]^

In the presented case, the patient refused to undergo programmed death-ligand 1 (PD-L1) testing for personal reasons. However, genomic profiling showed a high TMB. The prognosis of SMARCA4-UT may not be accurately predicted based solely on PD-L1 expression. Even cases with low or negative PD-L1 expression can demonstrate a lasting immunotherapy response.^[5,14]^ Several cases also indicated that PD-L1 expression was negative among the majority of SMARCA4-UT patients, who yet also presented with high TMB.^[14]^ Tumors characterized by high TMB may generate more neoantigens, thereby triggering a stronger antitumor immune response. Thus, TMB may function as a biomarker for forecasting a positive response to immune checkpoint inhibitors.^[27]^ According to a large-sample investigation, no significant association is found between PD-L1 expression and TMB across the majority of cancers, establishing them as independent biomarkers.^[28]^ Both have the potential to predict the immunotherapy efficacy, and their combined use enables more accurate prediction of the immune checkpoint inhibitor efficacy. In comparison to PD-L1 expression, the assessment of TMB is more stable and remains largely unaffected by the sampling time or treatment.^[29]^ Recently, Yang et al documented a case involving a SMARCA4-UT patient exhibiting a high TMB, who received a second-line treatment (tislelizumab plus etoposide and carboplatin), resulting in a tumor burden reduction that persisted for over 10 months.^[14]^ Dong et al presented a SMARCA4-UT patient experiencing a long-lasting response to tislelizumab plus etoposide and cisplatin, whose progression-free survival was more than 9 months.^[21]^ Due to economic reasons, tislelizumab plus etoposide and cisplatin is anticipated to emerge as a popular regimen for managing SMARCA4-UT. Mashell et al published a 62-year-old woman with SMARCA4-UT who received pembrolizumab, carboplatin, etoposide combined with radiotherapy, resulting in a 55% decreased size of her primary tumor.^[30]^

In this report, we endeavored to treat a SMARCA4-UT patient utilizing a first-line regimen comprising combination chemotherapy (carboplatin and etoposide), thoracic radiotherapy, and tislelizumab immunotherapy. The patient achieved a durable response. Drawing on multiple studies that underline the benefits of radiotherapy combined with immunotherapy,^[31–33]^ we administered immunotherapy after completing thoracic radiotherapy, which led to a long-term disease control (a progression-free survival of 16 months). In our case, the patient did not directly receive the chemotherapy combined with immunotherapy regimen at the beginning. Instead, after communication, he commenced immunotherapy subsequent to systemic chemotherapy and thoracic radiotherapy. This approach diverges somewhat from the previously reported treatment regimens. Firstly, systemic chemotherapy is administered to minimize the tumor volume and lower the risk of metastasis. Once the tumor is adequately controlled by chemotherapy, radiotherapy is implemented to reinforce the treatment. A combination of chemotherapy and radiotherapy is effective in killing tumor cells and releasing tumor antigens, which in turn amplifies the immunotherapy efficacy. The second-line treatment involved radiotherapy targeting the right adrenal gland metastasis, combination chemotherapy (paclitaxel and cisplatin), along with tislelizumab immunotherapy. The tumor cells in our male patient exhibited a high TMB, which may account for his prolonged response duration to tislelizumab immunotherapy. Compared with other analogous case reports, the OS of more than 33 months is considerably excellent. Drawing from experience reported in this case, the patient expresses hope that other SMARCA4-UT patients may also derive survival benefits.

## 4. Conclusion

In conclusion, no optimal standard protocol is available for managing SMARCA4-UT. Considering the constraints of individual case reports, the effectiveness of tislelizumab plus chemotherapy and radiotherapy warrants further validation in a more extensive cohort of individuals diagnosed with SMARCA4-UT.

## Acknowledgments

We feel grateful for the doctors and staff who have been involved in this work.

## Author contributions

**Conceptualization:** Rong Huang.

**Data curation:** Tao Zheng, Qingyu Ge.

**Investigation:** Xin Liang, Tao Zheng.

**Supervision:** Rong Huang.

**Writing – original draft:** Xin Liang, Tao Zheng, Rong Huang.

**Writing – review & editing:** Rong Huang.
